# Maternal Risk Factors for Stillbirth: A Registry–Based Study

**DOI:** 10.3390/medicina55070326

**Published:** 2019-07-01

**Authors:** Irisa Zile, Inguna Ebela, Ingrida Rumba-Rozenfelde

**Affiliations:** 1 Faculty of Medicine, Department of Paediatrica, University of Latvia, Raiņa bulvāris 19, LV-1050 Riga, Latvia; 2 The Centre for Disease Prevention and Control of Latvia, Duntes 22, k-5, LV-1005 Riga, Latvia

**Keywords:** stillbirth, maternal diseases, risk factors

## Abstract

*Background and Objectives:* The number of stillbirths has reduced more slowly than has maternal mortality or mortality in children younger than 5 years, which were explicitly targeted in the Millennium Development Goals. Placental pathologies and infection associated with preterm birth are linked to a substantial proportion of stillbirths. Appropriate preconception care and quality antenatal care that is accessible to all women has the potential to reduce stillbirth rates. The aim of the present study was to assess potential risk factors associated with stillbirth within maternal medical diseases and obstetric complications. *Materials and Methods:* Retrospective cohort study (2001–2014) was used to analyse data from the Medical Birth Register on stillbirth and live births as controls. Adjusted Odds ratios (aOR) with 95% confidence intervals (CI) were estimated. Multiple regression model adjusted for maternal age, parity and gestational age. *Results:* The stillbirth rate was 6.2 per 1000 live and stillbirths. The presence of maternal medical diseases greatly increased the risk of stillbirth including diabetes mellitus (aOR = 2.5; *p* < 0.001), chronic hypertension 3.1 (aOR = 3.1; *p* < 0.001) and oligohydromnios/polyhydromnios (aOR = 2.4; *p* < 0.001). Pregnancy complications such as intrauterine growth restriction (aOR = 2.2; *p* < 0.001) was important risk factor for stillbirth. Abruption was associated with a 2.8 odds of stillbirth. *Conclusions:* Risk factors most significantly associated with stillbirth include maternal history of chronic hypertension and abruptio placenta which is a common cause of death in stillbirth. Early identification of potential risk factors and appropriate perinatal management are important issues in the prevention of adverse fetal outcomes and preventive strategies need to focus on improving antenatal detection of fetal growth restriction.

## 1. Introduction

Stillbirths constitute an important worldwide problem that has generally received little attention. There are an estimated 2.6 million stillbirths each year, with 98% occurring in low- and middle-income countries [1]. The number of stillbirths has reduced more slowly than has maternal mortality or mortality in children younger than 5 years, which were explicitly targeted in the Millennium Development Goals [2]. Countries differ in their gestational age and birth-weight thresholds for stillbirth registration [3,4,5].

In Latvia, the stillbirth rate has no stable decrease tendency, in the last 10 years it is around 5 per 1000 births (5.6 in 2017). Stillbirth accounts for the largest proportion of perinatal deaths [6].

Numerous factors have been associated with an increased risk of stillbirth. Preventing stillbirth can be considered in terms of modifying risk factors, use of antenatal interventions, management of complications of pregnancy and the potential for population based screening for stillbirth [2,7,8,9,10,11,12,13]. However, the prevention of stillbirths has proved to be more challenging. The primary aim in this study was to assess potential risk factors associated with stillbirth within maternal medical diseases and obstetric complications.

## 2. Materials and Methods

This retrospective cohort study (2001–2014) analysed data from the Medical Birth Register (MBR). The study was conducted with the approval of the Ethics Committee of the University of Latvia, No.17/2014 (10 February 2014.). All births in Latvia (including stillbirths) are compulsorily reported to the registry, and notification is made by standardized medical record forms used by all maternity units across the country. Stillbirth were defined as fetal death occurring after 22 completed weeks of gestation and weighing at least 500 g. Data on live births was used as controls. In total, the data on 294,355 births was analysed.

The study analysed and compared maternal factors including maternal diseases, obstetric complications and antenatal care between still and live births. The analysis included factors related to the antenatal care (late first antenatal visit, i.e., registered to antenatal care and first visit after the 12th GW); maternal diseases (diabetes mellitus and gestational diabetes; chronic hypertension) and complications during pregnancy (antenatally detected intrauterine growth restriction (IGR), preeclampsia, rhesus isoimmunisation, pregnancy-induced hypertension, oligohydramnion /polyhydramnion, abruptio placenta).

Descriptive statistics for all the continuous variables are reported as mean ± standard deviation while categorical variables are reported as frequencies and percentages. The categorical variables were compared by using Chi-square test. Adjusted Odds ratios (aOR) with 95% confidence intervals (CI) were estimated. Logistic regression analyses were conducted to adjust the results for covariates or potential confounding variables such as maternal age, parity and gestational age. *P*-value ˂ 0.05 was considered as statistically significant.

Maternal age was coded as follows: ≤19 years, 20–34 years, and ≥35 years. Stillbirth period (2001–2014) rate were calculated per 1000 live and stillbirths.

The study was conducted with the approval of the Ethics Committee of the University of Latvia.

## 3. Results

There were a total of 1822 stillbirths and 292,533 live births during the period from 2001 to 2014 in Latvia. Totally an average is 20,000 births a year. The period (2001–2014) stillbirth rate was 6.2 (95% CI 5.9–6.5) per 1000 births. The heterogeneity in the events has observed and stillbirth rates are changing over the years. However, a slight decrease has been registered from 7.0/1000 (95% CI 5.9–8.2) births in 2001 to 5.2/1000 (95% CI 4.3–6.2) in 2014. There were 73.5% (95% CI 71.4–75.5%) of stillbirths that were antepartum. The results show that intrapartum stillbirths make quite a large proportion.

There were statistically significant differences in mean maternal age, gestational week and birth weight between still and live births. Mean maternal age for stillbirths was by 1.3 year (95% CI 1.1–1.6) higher and the proportion of mothers in age 35 years and more was by 8.7% points higher than for live births. Preterm births was more than half from stillbirths (66.0%). Within the stillbirths nullipara was observed by 5.8% points lower (*p* < 0.01) (Table 1).

Multiple births in stillbirths were by 4.7% points higher (*p* < 0.001) and the mothers who registered late for antenatal care was by 18.6% points higher (*p* < 0.001). Compared with the various maternal diseases, a higher proportion was observed in the stillbirths as well as complications during pregnancy (Table 1).

**Figure 1:**
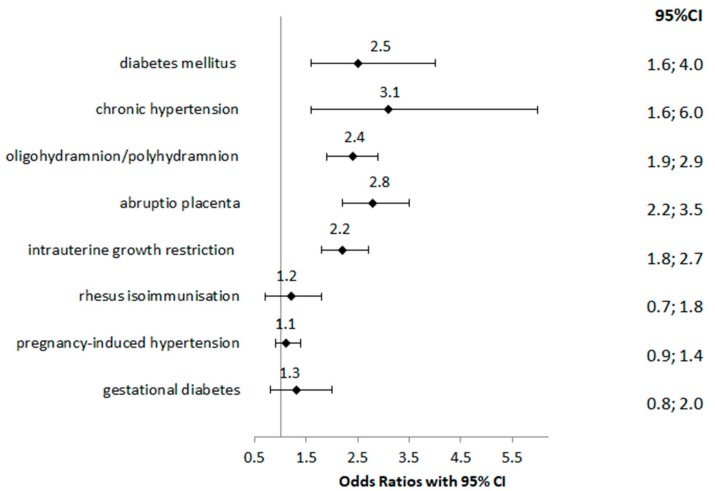
Maternal diseases and pregnancy complications associated with stillbirth (aOR; adjusted for maternal age, parity and gestational age and obtained from a logistic regression model).

The presence of maternal diseases increases the likelihood of developing a stillbirth during pregnancy or delivery, including: diabetes mellitus (aOR = 2.5; 95% CI 1.6–4.0; *p* < 0.001), chronic hypertension (aOR = 3.1; 95% CI 1.6–6.0; *p* < 0.001) and oligohydramnion/polyhydramnion (aOR = 2.4; 95% CI 1.9–2.9; *p* < 0.001). Pregnancy complications such as abruptio placenta (aOR = 2.8; 95% CI 2.2–3.5; *p* < 0.001) and intrauterine growth restriction (aOR = 2.2; 95% CI 1.8–2.7; *p* < 0.001) were associated with stillbirth. Pregnancy with Rhesus isoimmunisation, pregnancy-induced hypertension and gestational diabetes also had higher odds of stillbirth, but not in a statistically significant. Interpretation of the results should take into account frequency for each factor. For example the confidence interval of chronic hypertension is rather wide because the numbers of registered cases are very small. The wider the interval, the greater the uncertainty associated with our estimate (Figure 1). 

## 4. Discussion

The perinatal health monitoring system (PERISTAT) in Europe shows that the stillbirth and neonatal mortality rates continue to decline, albeit unevenly and more slowly than in previous periods [14]. In this study, the stillbirth proportion was 0.6% (2001–2014) and it was decreased from 0.7% to 0.5%. For example in England study stillbirth rate was 4.2 [12] which is lower than our mentioned (6.2/1000).

The intrapartum death rate of a country is reflective of the care received by mothers and babies in labour and rate higher than 10% indicates problems with obstetric care quality [15]. Substantial proportion of intrapartum stillbirths are preventable with quality intrapartum care and emergency obstetric care can make the greatest impact on stillbirth rates [2,15]. Quality intrapartum care is directly related to Millennium Development Goals—reduce child mortality and improve maternal health [16]. Improvement needs in Latvian healthcare system were documented also from international organizations (European Commission and the World Bank). For instance, there is a need to consider developing clinical guidelines and pathways based on clear criteria and standardized methods—improve quality of service provision, and coordination of services among healthcare providers and emerging legislation and regulatory frameworks [17]. Risk factors for stillbirth are increasingly being investigated and reported [8,9,10,11,12,13,14,18,19,20,21,22,23,24,25]. Meta-analysis and literature review revealed that maternal weight, maternal smoking, maternal age, primiparity, small size for gestational age, placental abruption and pre-existing maternal diabetes or hypertension were the most important and potentially modifiable risk factors [18].

In our study, stillbirths were also more frequently seen in women with multiple, preterm birth, women aged 35 years and with late first antenatal visit. The literature results also showed that inadequate antenatal care has also been associated with stillbirth, women who had their first antenatal visit after 20 weeks’ gestation were slightly more likely to have a stillborn baby than those who had earlier first visits (OR = 1.12) and odds more than tripled with no antenatal care [18].

This analysis focused on risk factors that would have been known at the start of pregnancy. Most of the associations found were consistent with the results of other studies. We identified important factors that contribute to stillbirth: diabetes mellitus [12,18,24], pre-existing hypertension [12,18,21], oligohydramnion/polyhydramnion, intrauterine growth restriction [10,12,13,22] and abruptio placenta [12,18,23].

Diabetes can have significant impacts on maternal, fetal and neonatal. The presence of diabetes can increase the risk of stillbirth by five times, and the risk of neonatal death by three times [18,24] which is similar to our findings (OR = 2.5). Meta-analysis of 70 studies on screening and management of diabetes during pregnancy indicates that the only timing intervention associated with a significant decrease in rate of stillbirths rates occurred during the preconception period [25].

The small number of registered cases of pre-pregnancy pathologies and pregnancy complications in MBR could affect result statistical significance. However, we do not believe that this alters our central findings or their clinical meaning. Prenatal control is very important and necessary treatment makes a positive and noticeable improvement in pregnancy outcome. Pre-existing hypertension remains an important risk factor for stillbirth and was associated with a rise in the odds of stillbirth of around three times which is also confirmed by other studies [18,21]. In this study, pregnancy-induced hypertension was not showed significant association with stillbirth. Opposite results observed in other study that the increased risk of stillbirth was higher in women having their second or higher order births (OR = 2.2) compared with women having their first birth [26]. Intrauterine growth restriction is a problem faced by obstetrical care providers on a daily basis [18,22] and we observed that it is increasing odds of stillbirth twice.

Opposite results were detected in a Japanese survey, that maternal complications and obstetric history showed no increase in the risk of stillbirth. Pregnancy induced hypertension and oligohydramnios even showed a reduced risk after adjusting for confounders. Researchers pointed that would apply to other relevant conditions, as these women are considered high-risk and followed up especially closely. This finding supports that better management of maternal conditions can reduce stillbirth in countries with higher stillbirth rates but functional obstetric care frameworks [10].

Placental abnormalities are a common cause of death in stillbirth, ranking second only to unexplained deaths [27,28,29]. This study results showed that placental abruption complicates 6% of pregnancies. Perinatal death is a devastating obstetric complication. Placental pathologies represent the largest category of cause of intrauterine death [28,29]. Determination of cause of death helps in understanding why and how it occurs. A perinatal death requires adequate diagnostic investigation. Histopathological examination of the placenta is recommended to determine the cause of stillbirth [28,29].

The limitation is the lack of detailed information about antenatal factors, life style habits, etc. Because the MBR includes just general information concerning antenatal and perinatal period factors not so detailed as it could be in clinical research using maternal medical history data. As well as some of the MBR entries could be incomplete for example about antenatally detected IGR. There is a possibility that not all data on maternal diseases are being reported in MBR. That is why in our study, we choose pathologies which are mostly should be reported in MBR such as hypertension, diabetes mellitus, etc. Special effort should be made to improve data reporting about all pre-pregnancy pathologies as well as different complications during pregnancy and delivery and create linkages between different databases for better epidemiological analysis in future.

Despite these limitations the main strength of the study includes the fact that the data were population-based which means that study utilized a large sample of women of reproductive age. Register-based data are essential for planning healthcare and determining temporal trends. PERISTAT also draws the attention that high quality health information is needed to support decision-making about health practices and policies for pregnant women and newborns [14].

Tools such as perinatal audits have been shown to improve the quality of facility care and to reduce stillbirths [7,14,19,20]. For example, PERISTAT data shows, the Netherlands and the UK implemented audits on stillbirths and report a greater reduction in stillbirth rates between 2010 and 2015 than other countries. More investigation of these case studies is needed to understand these relationships and could yield important examples of successful policy initiatives that could be adopted more widely [14]. Latvian Policy document “Maternal and Child Health Improvement Plan 2018–2020", developed by the Ministry of Health provides for improvements also in screening and maternal and perinatal mortality audit [30].

In order to decrease the rate of stillbirths among high risk mothers, a variety of interventions should been adopted. The keystone of these interventions has been early detection of maternal diseases (diabetes, hypertension, etc.) and where needed interventions to ‘normalize’ these so that complications can be reduced. With improvement in prenatal care, some of these deaths can be preventable.

## 5. Conclusions

The presence of maternal diseases increases the likelihood of developing a stillbirth during pregnancy or delivery. The risk factors most significantly associated with stillbirths include maternal history of chronic hypertension and diabetes mellitus. Pregnancies complicated by abruptio placenta have increased frequency of stillbirth. A placental pathology research can help better understanding of the association between placental abnormality and stillbirth.

Early identification of potential risk factors and appropriate perinatal management are important issues in the promotion of adverse fetal outcomes and preventive strategies need to focus on improving antenatal detection of fetal growth restriction.

## Figures and Tables

**Table 1:** Comparison of maternal characteristics and diseases in the stillbirths and live birth groups (2001–2014).

Characteristics	Stillbirths	Live Births	*p*-Value
	Mean (SD)	Mean (SD)	
Mean maternal age ^1^	28.8 (±6.4)	27.5 (±5.8)	<0.01
Mean gestational week ^1^	32.5 (±5.8)	39.1 (±1.8)	<0.001
Mean birth weight, g ^1^	1974.31 (±1113.0)	3456.71 (±568.4)	<0.001
	No (% (95CI))	No (% (95CI))	
Multiple births ^2^	127 (7.0 (5.9–8.2))	6843 (2.3 (2.2–2.4))	<0.001
Preterm births <37 GW ^2^	1203 (66.0 (63.8–68.2))	16115 (5.5 (5.4–5.6))	<0.001
Birth weight <2500 g ^2^	1193 (65.5 (63.3–67.6))	13838 (4.7 (4.6–4.8))	<0.001
*Maternal age* ^2^			
≤19 years	118 (6.5 (5.4–7.7))	22417 (7.6 (7.5–7.7))	<0.05
20–34 years	1306 (71.7 (69.6–73.7))	231694 (79.2 (79.1–79.4))	NS
≥35 years	398 (21.8 (20.0–23.8))	38422 (13.1 (13.0–13.3))	<0.001
Nullipara ^2^	789 (43.8 (41.5–46.1))	145029 (49.6 (49.4–49.8))	<0.01
First antenatal visit after 12 GW ^2^	483 (26.5 (24.5–28.6))	23009 (7.9 (7.7–8.0))	<0.001
Diabetes mellitus ^2^	21 (1.2 (0.7–1.7))	468 (0.2 (0.1–0.2))	<0.01
Gestational diabetes mellitus ^2^	19 (1.0 (0.6–1.6))	1767 (0.6 (0.5–0.6))	<0.05
Antenataly detected IGR ^2^	118 (6.5 (5.4–7.7))	3042 (1.0 (1.0–1.1))	<0.01
Abruptio placenta ^2^	98 (5.4 (4.4–6.5))	951 (0.3 (0.2–0.4))	<0.001
Preeclampsia ^2^	82 (4.5 (3.6–5.5))	6976 (2.4 (2.3–2.4))	<0.01
Rhesus isoimmunisation ^2^	20 (1.1 (0.7–1.7))	1535 (0.5 (0.4–0.6))	<0.01
Pregnancy-induced hypertension ^2^	86 (4.7 (3.8–5.8))	9574 (3.3 (3.2–3.4))	<0.01
Chronic hypertension ^2^	11 (0.6 (0.3–1.0))	271 (0.1 (0.08–0.1))	<0.01
Oligohydramnion/Polyhydramnion ^2^	93 (5.1 (4.1–6.2))	5398 (1.8 (1.7–1.9))	<0.01

^1^ Represents mean and standard deviation, T test is used; ^2^ Represents % (95% CI) and Chi square test is used; NS: Not Significant.
